# Scutellarin Protects Against Mitochondrial Reactive Oxygen Species-Dependent NLRP3 Inflammasome Activation to Attenuate Intervertebral Disc Degeneration

**DOI:** 10.3389/fbioe.2022.883118

**Published:** 2022-08-11

**Authors:** Zihao Wang, Pengfei Zhang, Yunpeng Zhao, Feiran Yu, Shaoyi Wang, Kaiwen Liu, Xiang Cheng, Jie Shi, Qiting He, Yanni Xia, Lei Cheng

**Affiliations:** ^1^ Department of Orthopaedic Surgery, Qilu Hospital, Cheeloo College of Medicine, Shandong University, Jinan, China; ^2^ Cheeloo College of Medicine, Shandong University, Jinan, China; ^3^ Qilu Hospital, Cheeloo College of Medicine, Shandong University, Jinan, China; ^4^ School of Medical Imaging, Weifang Medical University, Weifang, China

**Keywords:** intervertebral disc degeneration, scutellarin, mitochondrial ROS, NLRP3, NF-κB signaling 3 pathway

## Abstract

Intervertebral disc degeneration (IVDD) is a predominant cause of disc herniation and is widespread worldwide. Inflammatory responses, mitochondrial dysfunction, and extracellular matrix degradation are known to be involved in IVDD. Scutellarin, an active ingredient extracted from *Erigeron breviscapus* (Vaniot) Ha, *Hand-Mazz*, is reported to exhibit therapeutic potential in several degenerative diseases by suppressing inflammation and regulating metabolism. However, whether scutellarin can improve IVDD remains unknown. Human primary nucleus pulposus cells (HNPCs) were cultured and stimulated with TNF-α in the presence or absence of scutellarin. Furthermore, a rat needle puncture model was established, and scutellarin was injected into the IVD to verify its protective function against IVDD. Scutellarin attenuated the inflammatory reaction and retained the production of major IVD components both *in vitro* and *in vivo*. Mechanistically, scutellarin reduced the amount of reactive oxygen species (ROS), alleviated mitochondrial damage, and decreased the expression levels of apoptosis-related biomarkers upon stimulation with TNF-α. In addition, scutellarin antagonized the activation of the nuclear factor κ-light-chain-enhancer of activated B (NF-κB) signaling pathway and the mitogen-activated protein kinase (MAPK) signaling pathway and suppressed the activity of the NLRP3 inflammasome mediated by TNF-α. This study reveals that scutellarin protects against degeneration of nucleus pulposus cells, which might shed light on treatment of IVDD in the future.

## Introduction

To date, more than 80% of people all over the world suffer from low back pain (LBP), and 10% of these people would develop chronic disability (Knezevic et al., 2021; Chen et al., 2022). Recent studies have shown that LBP is closely associated with intervertebral disc degeneration (IVDD) by interfering with the structure and function of the intervertebral disc (IVD) (Novais et al., 2021). Reactive oxygen species (ROS), oxidative stress, and mitochondrial dysfunction are well accepted to be involved in the pathogenesis of IVDD (Zhao et al., 2020; Hu et al., 2021; Yu et al., 2021). ROS exaggerate various pathological processes in IVDD development, such as elevating inflammatory cytokine production and enhancing apoptosis in IVD cells (Wang B. et al., 2021; Wang Y. et al., 2021). Mitochondria are a main target of ROS. Intriguingly, mitochondrial dysfunction is a predominant cause of ROS production (Kang et al., 2020). High levels of ROS lead to the destruction of mitochondrial function, which results in excessive catabolism in nucleus pulposus (NP) cells and increases inflammation in the microenvironment of the IVD (Xia et al., 2019).

The inflammatory microenvironment in the nucleus pulposus (NP) is well established to be associated with IVDD, and TNF-α plays a critical role during this process (Zhao et al., 2020). TNF-α can reduce the levels of aggrecan and type II collagen (Col-2) and exacerbate the degradation of the extracellular matrix (ECM) by enhancing the expression of matrix metalloproteinases (MMPs), as well as a disintegrin and metalloproteinase with thrombospondin motifs (ADAMTSs) (Liao et al., 2021; Zhu et al., 2021). Furthermore, TNF-α is reported to elevate ROS production and the release of downstream inflammatory cytokines, which potentially enhance cell apoptosis and senescence (Sun et al., 2020). These pathological alterations caused by TNF-α consequently lead to the formation of an inflammatory microenvironment in NP tissue and the development of IVDD. Among the downstream signaling pathways involved in TNF-α induction of disorganized cell function, the NF-κB and MAPK signaling pathways are closely associated with degeneration of IVD and are extensively studied as potential therapeutic targets for IVDD.

To date, the NACHT, LRR, and PYD domain-containing protein 3 (NLRP3) inflammasome is closely associated with inflammation (Magupalli et al., 2020) and plays a detrimental role in the degeneration of several systems (Tarallo et al., 2012; Fowler et al., 2014). Activation of the NLRP3 inflammasome leads to the production of IL-1β and IL-18, which subsequently leads to disordered metabolism and impaired cell viability (Hofbauer et al., 2021). Recent studies have reported that the NLRP3 inflammasome is a contributor to the disorganization of homeostasis, and activation of the NLRP3 inflammasome is closely associated with IVDD (Song et al., 2017; Zhao et al., 2021).

Scutellarin (4′,5,6-hydroxyflavone-7-glucuronide), a flavonoid glucuronide isolated from *Erigeron breviscapus Hand.-Mazz.*, has been shown to have a wide range of pharmacological activity, including anti-inflammatory, antioxidant, antidiabetic and antihyperlipidemic properties (Wang et al., 2020). Scutellarin suppresses oxidative stress as well as mitochondrial dysfunction in several pathological processes (Bian et al., 2020; Xi et al., 2021). Scutellarin attenuates artificially induced apoptosis as well as ECM degradation associated with arthritis (Wang et al., 2019). Scutellarin has been reported to probably mediate such effects by inhibiting inflammatory cytokine expression by dampening inflammatory signaling pathways, including the NF-κB signaling pathway (Wang et al., 2019) and the MAPK signaling pathway, including p38 (Liu et al., 2020) and c-Jun N-terminal kinase (JNK) (Chen et al., 2020). Moreover, scutellarin can suppress activation of the NLRP3 inflammasome and attenuate the inflammatory reaction and apoptosis (Li et al., 2020). Nevertheless, the role of scutellarin in the development of IVDD remains to be elucidated. In this study, we plan to explore whether scutellarin protects against IVDD and, if so, the potential mechanisms involved.

## Materials and Methods

### Ethics Statement

In this study, human IVD tissues were acquired from 19 patients (10 males and 9 females; 18–35 years old) with IVDD (all with grade II IVDD) who underwent lumbar spine surgery at Qilu Hospital of Shandong University. The degree of IVDD was assessed using the modified Pfirrmann grading system (Pfirrmann et al., 2001). Collection of lumbar disc tissue samples adhered to medical ethics regulations and was approved by the Medical Ethical Committee of Qilu Hospital of Shandong University with the ID of KYll-2021 (ZM)-058. A written informed consent document was requested and received from all study participants. All animal experimental procedures were performed according to the International Guiding Principles for Animal Research and were approved by the Laboratory Animal Centre of Shandong University.

### Isolation and Culture of Human Enucleus Pulposus Cells

NP cells were isolated and cultured as previously reported (Zhao et al., 2020; Yu et al., 2021). After the human IVD samples were washed 3 times with phosphate-buffered saline (PBS), the endplate cartilage and annulus fibrosus were removed. Then, the IVD tissues were carefully cut into fragments of approximately 1 mm^3^ in volume and digested with 0.25% trypsin and collagenase II (Sigma, United States). Then, the isolated cells were cultured in Dulbecco’s modified Eagle medium (DMEM)/F12 (HyClone, United States) supplemented with 10% fetal bovine serum (FBS) (Gibco, United States) and 1% penicillin and streptomycin (P1400, Solarbio, China) under standard incubation conditions (37°C, 5% CO_2_, 95% air). The cell culture medium was replaced every 3 days, and the cells were passaged when they reached 80–90% density. HNPCs from passages 2–5 were used for further experiments. HNPCs were harvested and cultured in 6-well, 12-well, and 24-well plates and treated with or without TNF-α (HY-P7058, MCE, United States) (10 ng/ml) in the absence or presence of scutellarin (HY-N0751, MCE) (1 μM) for 15 min, 30 min, 1 h, 6 h, 24 h or 48 h before use in various experiments.

### Rat Model Establishment

We purchased eight-week-old male Sprague Dawley rats from Beijing Vital River Laboratory Animal Technology Co., Ltd. The rats were housed under controlled identical specific pathogen-free (SPF) standard environmental conditions (23 ± 2°C, 12-h light/dark cycle) with free access to food and allowed to move freely. The IVDD model was established as previously reported (Zhao et al., 2020; Wu et al., 2021; Yu et al., 2021). Briefly, 15 rats were anesthetized by intraperitoneal injection with 2% (w/v) pentobarbital (40 mg/kg). As shown in Figure 3 of Supplementary Material, after being located by the X-ray and micro CT, the rat 6/7, 7/8, and 8/9 coccygeal discs were punctured using a 21 G needle. The depth of puncture was controlled at 5 mm. Later, the needles were rotated 360° and kept in position for 1 min. Then, 2.5 μl of PBS or scutellarin (100 ng/μl) was injected into the rat coccygeal discs using a microinjector (Hamilton, United States) with a 30 G needle (*n* = 5). After the aforementioned surgical procedure, the rats were monitored daily to observe their health condition.

### X-Ray and Magnetic Resonance Imaging

To evaluate the structural differences and signal intensity changes in sagittal T2-weighted images of NPs, the rats in each group underwent X-ray and MRI scanning before execution 14 days after the initial puncture. The rats were placed in the prone position with their tails outstretched on the molybdenum target radiographic-image unit (GE, Boston, MA, United States). Radiographs were captured at a collimator-to-film distance of 66 cm, an exposure of 63 mAs, and a penetration power of 35 kV. T2-weighted images (repetition time: 3,000 ms; echo time: 80 ms; field of view: 200 mm^2^; slice thickness: 1.4 mm) were obtained by magnetic resonance imaging (MRI) using a 1.5 T system (GE) in the coronal plane. All radiographic images were saved in a Neusoft PACS/RIS DICOM 3.0 medical imaging system (Neusoft, Jinan, China). The IVD height and the adjacent upper and lower vertebral body heights were measured using ImageJ software, and the disc height index (DHI) was calculated from these values. The MRI grade of NPs was evaluated as previously reported (Xu et al., 2019; Zhao et al., 2020).

### Real-Time Polymerase Chain Reaction

An RNAfast200 Kit (220011, Fastagen, China) was used to extract total RNA from the HNPCs according to the recommended procedure. Complementary cDNA was synthesized using a cDNA synthesis kit (FSQ-101, Toyobo, Japan). Real-time PCR was carried out with SYBR Green PCR Master Mix (OPK-201, Toyobo). For each sample, the relative mRNA levels were normalized to the group treated with PBS. The expression levels of target genes were normalized to GAPDH. The specific PCR products for each gene were confirmed by performing a melting curve analysis. The experiment was repeated three times for each group and each target gene. The sequence-specific primers used for real-time PCR are listed in Table 1. The results were calculated using the 2^−ΔΔCt^ method and are expressed as the fold change.

**TABLE 1:** Primers used for quantitative real-time PCR.

Primer	Forward primers, 5′–3′	Reverse primers, 5′–3′
Homo-ADAMTS5	GAA​ACA​ACG​GAC​GCT​ACT​GC	ATG​ATT​TAC​CAT​TGG​GTG​GGC​A
Homo-MMP-13	ATT​AAG​GAG​CAT​GGC​GAC​TTC​T	GCC​CAG​GAG​GAA​AAG​CAT​GA
Homo-aggrecan	GGT​CTC​ACT​GCC​CAA​CTA​CC	CAC​GAT​GCC​TTT​CAC​CAC​GA
Homo-COL2	GAT​GGC​TGC​ACG​AAA​CAT​ACC	GCC​CTA​TGT​CCA​CAC​CGA​AT
Homo-INOS	CGT​GGA​GAC​GGG​AAA​GAA​GT	GAC​CCC​AGG​CAA​GAT​TTG​GA
Homo-COX2	TCC​CTT​GGG​TGT​CAA​AGG​TAA​A	TGG​CCC​TCG​CTT​ATG​ATC​TG
Homo-CASP3	AAA​AGC​ACT​GGA​ATG​ACA​TCT​CGG	TGG​CTC​AGA​AGC​ACA​CAA​ACA
Homo-BCL2	GGG​TGA​ACT​GGG​GGA​GGA​TT	ATC​TCC​CGG​TTG​ACG​CTC​TC
Homo-BAX	GAG​GTC​TTT​TTC​CGA​GTG​GCA	GGC​AAA​GTA​GAA​AAG​GGC​GAC
Homo-NLRP3	GAG​CCG​AAG​TGG​GGT​TCA​GA	CTT​CAA​TGC​TGT​CTT​CCT​GGC
Homo-IL-1β	CAA​CAA​GTG​GTG​TTC​TCC​ATG​TC	ACA​CGC​AGG​ACA​GGT​ACA​GA
Homo GAPDH	GCA​CCG​TCA​AGG​CTG​AGA​AC	TGG​TGA​AGA​CGC​CAG​TGG​A

### Western Blotting

HNPCs were cultured using the methods described above to detect different indicators by Western blotting. Total proteins were extracted from HNPCs from each treated group with precooled radioimmunoprecipitation assay (RIPA) lysis buffer (P0013C, Beyotime Biotechnology, China) containing 1 μM phenylmethylsulfonyl fluoride (PMSF) with or without phosphatase inhibitors. The protein concentration was detected with a bicinchoninic acid (BCA) protein assay kit (PC0020, Solarbio). Equal amounts of protein from each sample were separated by sodium dodecyl sulfate–polyacrylamide gel electrophoresis (SDS-PAGE) on 8%, 10%, or 12% SDS-polyacrylamide gels and then transferred to a polyvinylidene difluoride (PVDF) membrane (Millipore, United States). After blocking with QuickBlockTM Blocking Buffer (P0252, Beyotime Biotechnology) for 20 min at room temperature, the membranes were incubated with rabbit primary antibodies against iNOS(1:1000, 18985-1-AP, Proteintech, United States), COX-2 (1:1000, ab15191, Abcam, United States), GAPDH (1:5000, 10494-1-AP, Proteintech), Aggrecan (1:1000, 13880-1-AP, Proteintech), Col-2 (1:1000, 28459-1-AP, Proteintech), ADAMTs-5 (1:1000, ab41037, Abcam), MMP-13 (1:1000, sc-515284, Santa Cruz Biotechnology), Bcl-2 (1:1000, ab196495, Abcam), Bax (1:1000, BM3964, Boster, China), Cleaved caspase-3 (1:1000, AF7022, Proteintech), OPA1(1:1000, PB0773, Boster), Drp1 (1:1000, 12957-1-AP, Proteintech), Mfn1 (1:1000, 13798-1-AP, Affinity, China), Mfn2 (1:1000 12186-1-AP, Proteintech), NLRP3 (1:1000, ab214185, Abcam), p-p65 (1:1000, 8242T, Cell Signaling Technology, United States), p65 (1:1000, 3033S, Cell Signaling Technology), p-p38 (1:1000, 4511T, Cell Signaling Technology), p38 (1:1000, 8690T, Cell Signaling Technology), p-JNK (4688S, Cell Signaling Technology), JNK (1:1000, 9252T, Cell Signaling Technology), p-Erk (1:1000, AF1015, Affinity), and Erk (1:1000, 4695T, Cell Signaling Technology) overnight at 4°C. Then, the membranes were incubated for 90 min at room temperature with a secondary antibody. The bands were visualized using an Amersham Imager 600, and the density of protein bands was quantified using ImageJ software.

### Immunohistochemistry

The rats were sacrificed 2 weeks after the indicated surgery, and IVD tissues were collected. After fixation in 4% paraformaldehyde, the IVD tissues were decalcified, dehydrated, cleared using dimethylbenzene, and embedded in paraffin. Then, they were cut into 5-μm sections. Immunohistochemistry was performed with an Immunohistochemistry Kit (SP-9000, ZSGB-BIO, China) according to the instructions. After dewaxing and hydration, the sections were treated with Tris-ethylenediaminetetraacetic acid (EDTA) antigen retrieval buffer (C1038, Solarbio) for 10 min at 95°C and endogenous peroxidase blocker for 10 min. After blocking in goat serum for 30 min at room temperature, the cells were incubated with primary antibodies against iNOS (1:200, 18985-1-AP, Proteintech), COX-2 (1:200, ab15191, Abcam), ADAMTS-5 (1:200, ab41037, Abcam), MMP-13 (1:200, sc-515284, Santa Cruz Biotechnology, China), NLRP3 (1:200, Affinity, DF7438) at 4°C overnight. Then, the sections were incubated with goat antirabbit immunoglobulin (IgG) secondary antibodies for 1 h at room temperature and horseradish peroxidase (HRP)-labeled *Streptomyces* ovalbumin for 15 min. Detection was performed by using the DAB Substrate kit (ZLI-9018, ZSGB). Then, the slides were counterstained with 1% hematoxylin. Images were captured by a microscope (Leica DMI3000B). The positive areas were quantified using ImageJ.

### Immunofluorescence_staining

Treated HNPCs on coverslips were fixed with 4% paraformaldehyde for 30 min. After incubation with 0.1% Triton X-100 for 5 min, the samples were blocked with 5% bovine serum albumin (BSA) at 37°C for 1 h. Next, the samples were incubated with primary antibodies against iNOS (1:1000, 18985-1-AP, Proteintech), COX-2 (1:1000, ab15191, Abcam), Col-2 (1:1000, 28459-1-AP, Proteintech), MMP-13 (1:1000, sc-515284, Santa Cruz Biotechnology), and p65 (1:1000, AF5008, Affinity) at 4°C overnight and then with fluorescently labeled goat anti-IgG (A23220, Amyjet Scientific or A23320, Amyjet Scientific) for 1 h at 37°C. The nuclei were stained with diamidino-2-phenylindole (DAPI).

The IVD sections were dewaxed and dehydrated, and the sections were treated with Tris-EDTA antigen retrieval buffer (C1038, Solarbio) for 10 min at 95°C. Next, the sections were incubated with 0.1% Triton X-100 for 5 min and 5% BSA at 37°C for 1 h. Then, the tissue sections were incubated with primary antibodies against cleaved caspase-3 (1:100, AF7022, Affinity) at 4°C overnight. The sections were incubated with fluorescently labeled goat anti-IgG (A23220, Amyjet Scientific or A23320, Amyjet Scientific) for 1 h at 37°C, and the nuclei were stained with DAPI.

The coverslips and sections were then observed by a fluorescence microscope (ZEISS Vert. A1), and the immunofluorescence signal intensities were quantified with ImageJ software.

### Enzyme-Linked Immunosorbent Assay

The cell culture supernatants of each group were collected and preserved at −80°C until detection. Then, the IL-1β secretory expression levels were detected by an ELISA kit (E-EL-H0149c, Elabscience, China) according to the manufacturer’s instructions with a microplate reader (Infinite M NANO, TECAN).

### MitoTracker Assay

MitoTracker Red CMXRos (C1049B, Beyotime Biotechnology) was used to detect mitochondrial activity and membrane potential. According to the manufacturer’s instructions, the cells were incubated with a culture medium containing 20 nM MitoTracker Red CMXRos for 30 min at 37°C in the dark and then observed and captured with a fluorescence microscope (ZEISS Vert. A1) after changing the fresh culture medium.

### JC-1 Assay

The mitochondrial membrane potential was detected using a JC-1 assay kit (C2003S, Beyotime Biotechnology). Treated HNPCs in 6-well plates were stained with a JC-1 staining solution at 37°C for 20 min in the dark according to the manufacturer’s instructions. Then, the cells were washed twice with 1× JC-1 staining buffer, and the images were observed and captured using a fluorescence microscope (ZEISS Vert. A1).

### Adensine Triphosphate Production Assay

The ATP Bioluminescence Assay kit (S0026, Beyotime Biotechnology) was used to quantify intracellular ATP production. ATPs were extracted from cells treated with ATP for lysis. All procedures were performed according to the manufacturer’s instructions with a luminometer (Centro XS3 LB 960, Berthold Technologies).

### Terminaldeoxynucleotidyl Transferase dUTP Nick End Labelling (TUNEL) Staining

Human NP cells on coverslips were stained with the TUNEL assay Kit (E-CK-A331, Elabscience) to examine the cellular apoptosis of each experimental group. All procedures were performed in accordance with the manufacturer’s instructions. The images were captured using a fluorescence microscope (ZEISS Vert. A1).

### Reactive Oxygen Species Assay

Intracellular ROS levels were detected with a ROS assay kit (S0033S, Beyotime Biotechnology). Briefly, after washing twice with sterile PBS, HNPCs were stained with 10 μM 2′,7′-dichlorofluorescein diacetate (DCFDA) at 37°C for 20 min in the dark and mixed every 5 min. Then, the HNPCs were washed three times with a basal culture medium before being observed and captured using a fluorescence microscope (ZEISS Vert. A1).

### Histological Staining and Assessment

The rats were sacrificed 2 weeks after surgery. The IVD tissues were collected and fixed in 4% paraformaldehyde. After decalcification, dehydration, and embedding in paraffin, the IVDs were cut into 5-μm sections. Hematoxylin and eosin (H & E) staining was performed to evaluate the morphological changes of NPs with an H & E staining Kit (EE0012, Sparkjade), while Safranin O-Fast green staining was also performed to examine the cellularity and morphology of NPs and AF with a Modified Safranine O-Fast Green FCF Cartilage Stain Kit (G1371, Solarbio). The images were captured by a microscope (Leica DMI3000B).

For H&E staining, we evaluated histological grading of the IVD samples in accordance with the grading scale, which is based on the morphology of the AF and the cellularity of the NP (Keorochana et al., 2010). For safranin O-fast green staining, we conducted histological grading of the IVD samples according to the grading scale previously published (Han et al., 2008). The grading scale is based mainly on the cellularity and morphology of the AF and NP as well as the border between the two structures. The grades range from 5–15. The higher the score is, the more severe the degeneration.

### Transmission Electron Microscopy

HNPCs were collected by trypsinization, transferred into 2-ml centrifuge tubes, and fixed with fixation solution (G1102, Servicebio, China) for 2 h at 4°C in the dark. Then, the cells were proembedded with the agarose solution and postfixed in 1% osmium tetroxide in 0.1 M phosphate buffer (pH 7.4). Then, the cells were dehydrated in a graded series of ethanol solutions (30%, 50%, 70%, 80%, 95%, and 100%) for 15 min for each solution and infiltrated with a propylene oxide embedding medium overnight. Ultrathin sections (50 nm) were obtained using a Leica UC7 ultramicrotome, poststained with uranyl acetate and lead citrate, and visualized using a TEM (HT7800/HT7700).

### Flow Cytometry

The apoptosis of HNPCs from each experimental group was detected by flow cytometry. Cells were collected into flow cytometry tubes and stained with propidium iodide (PI) and Annexin V-FITC for 15 min at room temperature away from light with a fluorescein isothiocyanate (FITC) Annexin V Apoptosis Detection Kit (E-CK-A211, Elabscience) according to the manufacturer’s instructions. Cell apoptosis was detected with a CytoFLEX S flow cytometer (Beckman Coulter, United States), and the data obtained were analyzed with CtyExpert software.

### Statistical Analysis

The blinded method was conducted in all data acquisitions. Data are presented as the means ± SD of results derived from three independent experiments performed in triplicate. Analysis of data was performed with GraphPad Prism software (version 7.0; GraphPad Inc., La Jolla, CA, United States). Comparisons of different treatment groups were performed using analysis of variance (ANOVA) with Tukey’s post-hoc test. Statistical significance was considered when *p* < 0.05.

## Results

### Scutellarin Alleviated the TNF-α-Induced Inflammatory Response in Primary Human Nucleus Pulposus Cells

**FIGURE 1:**
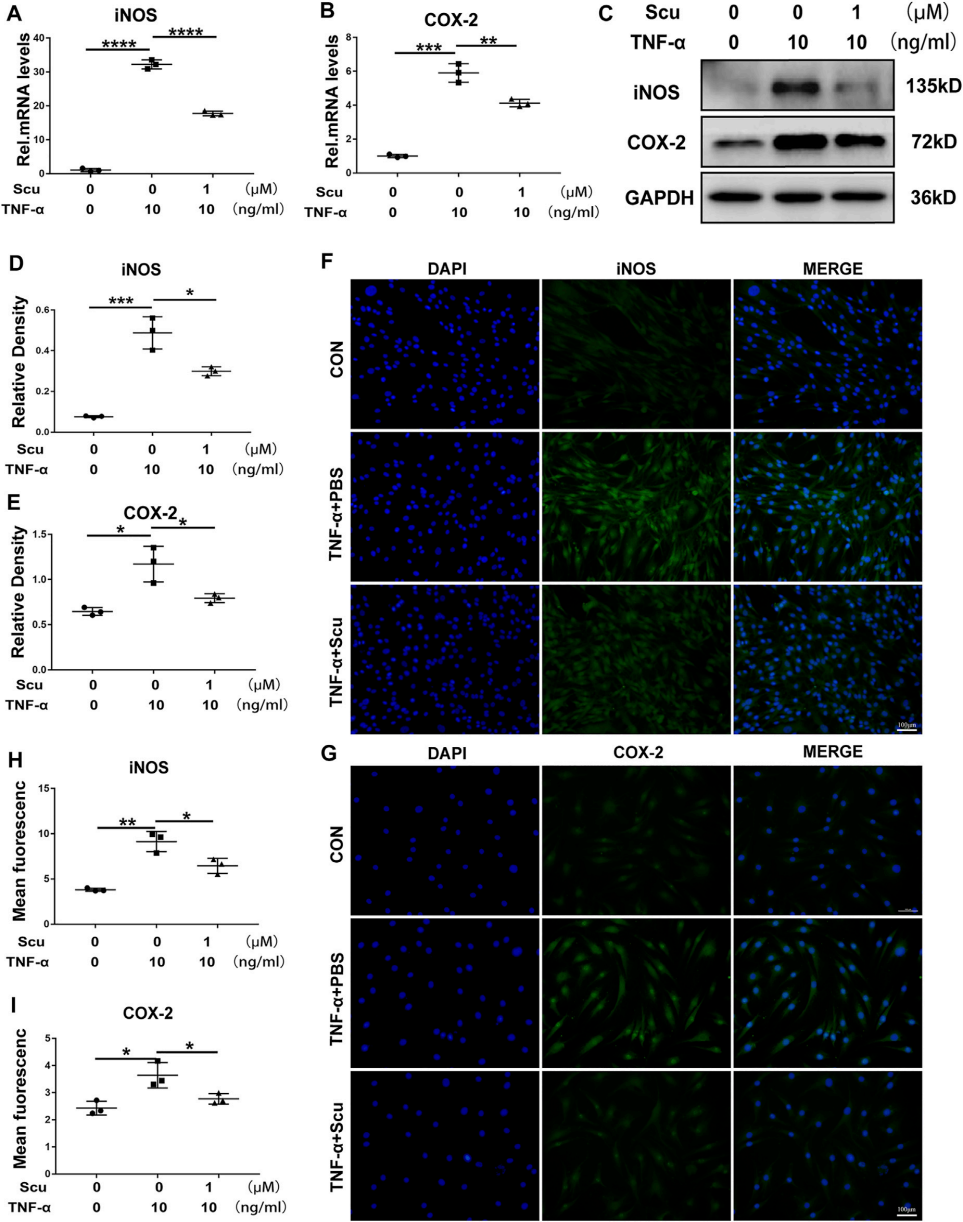
Scutellarin alleviated the TNF-α-induced inflammatory response in HNPCs. **(A,B)** HNPCs were treated with or without TNF-α (10 ng/ml) in the absence or presence of scutellarin (1 μM) for 24 h. Total RNA from each group was extracted, and real-time PCR was performed to examine the mRNA expression of iNOS and COX-2. **(C–E)** HNPCs were treated with PBS, TNF-α (10 ng/ml), TNF-α (10 ng/ml) +scutellarin (1 μM) for 48 h. The protein extractions of each group were detected by Western blotting with anti-iNOS and antiCOX-2 antibodies. **(F–I)** HNPCs were cultured as indicated for 48 h. Immunofluorescence cell staining was performed to detect the expression of iNOS and COX-2 (scale bar: 100 μm). Each experiment was performed three times independently. (*p < 0.05, **p < 0.01, ***p < 0.001, ****p < 0.0001).

Previous studies have shown that elevated expression of inflammatory cytokines is a critical factor leading to IVDD (Liu K. et al., 2021). To investigate the role of scutellarin in the progression of the inflammatory response during IVDD, primary human nucleus pulposus cells (HNPCs) were isolated and then cultured with TNF-α in the presence or absence of scutellarin for 24 h to detect the mRNA level and 48 h to detect the protein level of inflammatory biomarkers. Following the indicated treatments, mRNA was extracted from the HNPCs, and real-time PCR was performed. Figures 1A,B show that the expression levels of COX-2 and iNOS were increased after TNF-α stimulation, while administration of scutellarin decreased the production of these cytokines induced by TNF-α. Next, total protein was extracted from each group of HNPCs, and Western blotting was performed to detect the expression levels of COX-2 and iNOS. As shown in Figures 1C–E, TNF-α-mediated enhancement of inflammatory cytokines was repressed by additional treatment with scutellarin. In the present study, immunofluorescence staining was performed for COX-2 and iNOS, indicating that the TNF-α-induced inflammatory reaction of HNPCs was markedly attenuated by scutellarin treatment (Figures 1F–I).

### Scutellarin Retained Cell Metabolism and Alleviated Degeneration of Primary Human Primary Nucleus Pulposus Cells

**FIGURE 2:**
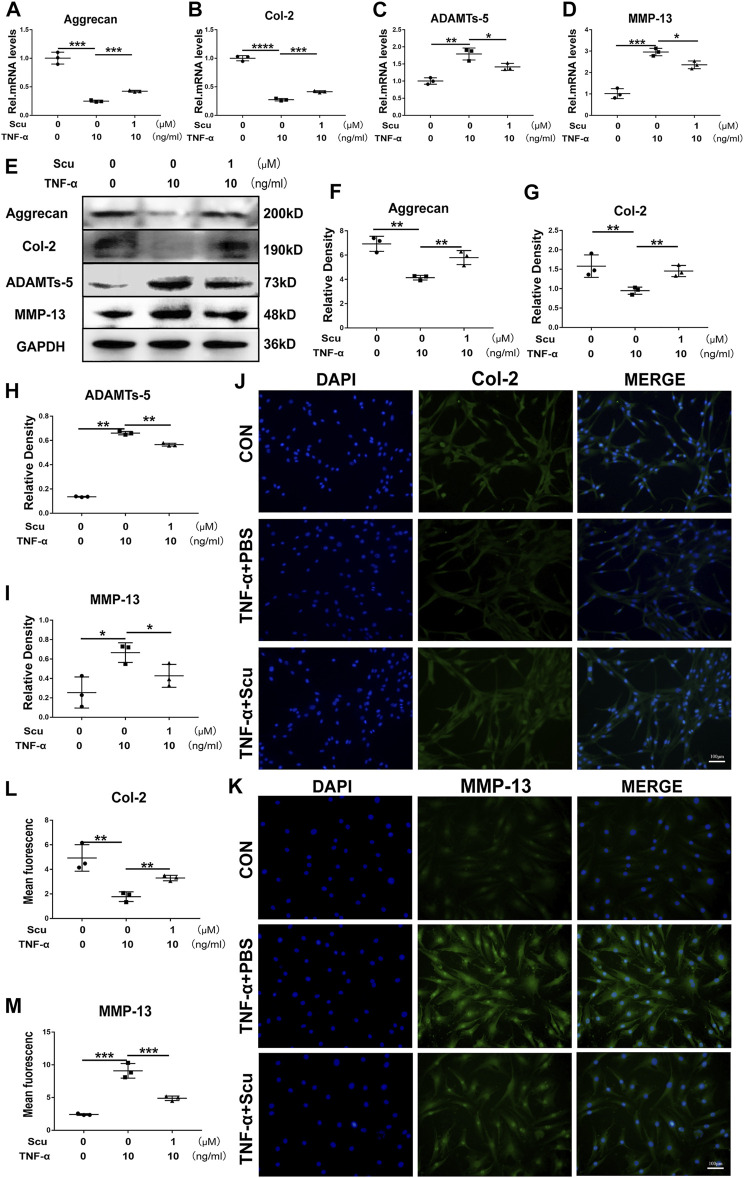
Scutellarin retained cell metabolism and alleviated degeneration of primary HNPCs. **(A–D)** HNPCs were treated with or without TNF-α (10 ng/ml) in the absence or presence of scutellarin (1 μM) for 24 h. Then, total RNA from each group was extracted, and the transcriptional expression of Aggrecan, Col-2, ADAMTS-5, and MMP-13 was measured by real-time PCR. **(E–I)** HNPCs were cultured with PBS, TNF-α (10 ng/ml), TNF-α (10 ng/ml) +scutellarin (1 μM) for 48 h. The protein expression levels of Aggrecan, Col-2, ADAMTS-5, and MMP-13 were assayed by Western blotting. **(J–M)** After culturing as indicated for 48 h, immunofluorescence staining was performed to examine the protein levels of Col-2 and MMP-13 (scale bar: 100 μm). Each experiment was performed three times independently. (*p < 0.05, **p < 0.01, ***p < 0.001).

TNF-α enhances the expression of ADAMTSs and MMPs, leading to a reduction in ECM synthesis and the exaggeration of ECM degradation, among which MMP-13 and ADAMTS-5 play a critical role in Col-2 and Aggrecan levels (Fu et al., 2021). In this study, primary HNPCs were cultured and stimulated with TNF-α with or without scutellarin treatment. At the 24 h time point, mRNA was collected from each group, and real-time PCR was performed. The mRNA levels of the anabolic biomarkers Aggrecan and Col-2 were diminished, while the catabolic factors MMP-13 and ADAMTS-5 were elevated following stimulation with TNF-α. Nevertheless, additional treatment with scutellarin displayed a protective effect in the presence of TNF-α (Figures 2A–D). Moreover, total protein was isolated from HNPCs after the indicated treatment for 48 h, and the levels of Aggrecan, Col-2, MMP-13, and ADAMTS-5 were detected through Western blotting. As shown in Figures 2E–I, TNF-α stimulation decreased Aggrecan and Col-2 and promoted MMP-13 and ADAMTS-5 expression, while scutellarin antagonized the impairment of metabolism in HNPCs induced by TNF-α. These results were further verified by immunofluorescence staining, as shown in Figures 2J–M. TNF-α-induced reduction of Col-2 and elevation of MMP-13 signals were reversed by scutellarin administration.

### Scutellarin Treatment Suppressed TNF-α-Mediated Apoptosis in Human Primary Nucleus Pulposus Cells

**FIGURE 3:**
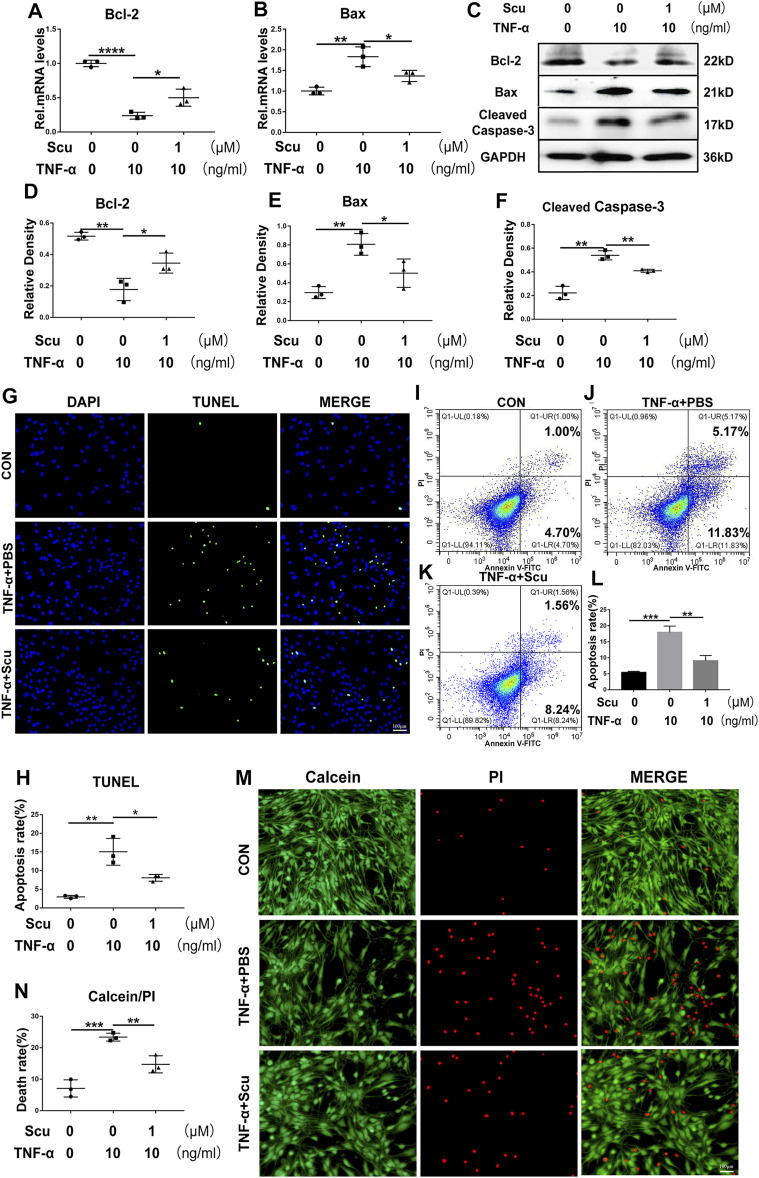
Scutellarin treatment suppressed TNF-α-mediated apoptosis in HNPCs. **(A,B)** HNPCs were stimulated with PBS, TNF-α (10 ng/ml), TNF-α (10 ng/ml) +scutellarin (1 μM) for 24 h. Total RNA was extracted from each group, and the mRNA expression of Bcl-2 and Bax was assayed using real-time PCR. **(C–F)** HNPCs were treated as indicated for 48 h. Total protein was extracted from each group of HNPCs, and the protein expression levels of Bcl-2, Bax, and cleaved caspase-3 were assayed using Western blotting. **(G,H)** TUNEL staining for each indicated group to examine the apoptosis of HNPCs (scale bar: 100 μm). **(I–L)** Apoptosis of each indicated group was detected by flow cytometry after treatment as indicated for 48 h. **(M,N)** Calcein/PI staining was performed to detect the death of HNPCs treated as indicated for 48 h (scale bar: 100 μm). Each experiment was performed three times independently. (*p < 0.05, **p < 0.01, ***p < 0.001).

Apoptosis of nucleus pulposus cells has been reported to be a critical factor associated with IVDD (Li BL. et al., 2021). To demonstrate the function of scutellarin in apoptosis of HNPCs, the cells were cultured with TNF-α in the presence or absence of scutellarin for 24 h to detect the mRNA levels of apoptosis-associated molecules. As shown in Figures 3A,B, Bcl-2 levels were reduced, and Bax levels were increased after stimulation with TNF-α. Scutellarin reversed the alteration of these biomarkers induced by TNF-α. Moreover, the cells were subjected to the indicated treatments for 48 h, and total protein was extracted from each group of HNPCs. Western blotting results indicated that TNF-α-mediated disorganization of these apoptosis biomarkers, including reduction of Bcl-2 and elevation of Bax as well as cleaved caspase-3, was attenuated by scutellarin treatment (Figures 3C–F). To further determine the role of scutellarin in apoptosis of HNPCs, TUNEL staining (Figures 3G,H) and flow cytometry (Figures 3I–L) were performed, which revealed that TNF-α promoted cell apoptosis, while scutellarin repressed this disorganization. Additionally, a live/dead assay was performed to verify the apoptosis rate of HNPCs, which indicated that TNF-α exaggerated the death of HNPCs, which was markedly diminished by scutellarin treatment (Figures 3M,N).

### Scutellarin Reverses Oxidative Stress and Mitochondrial Dysfunction and Reduced Adensine Triphosphate Production Induced by TNF-α in Human Primary Nucleus Pulposus Cells

**FIGURE 4:**
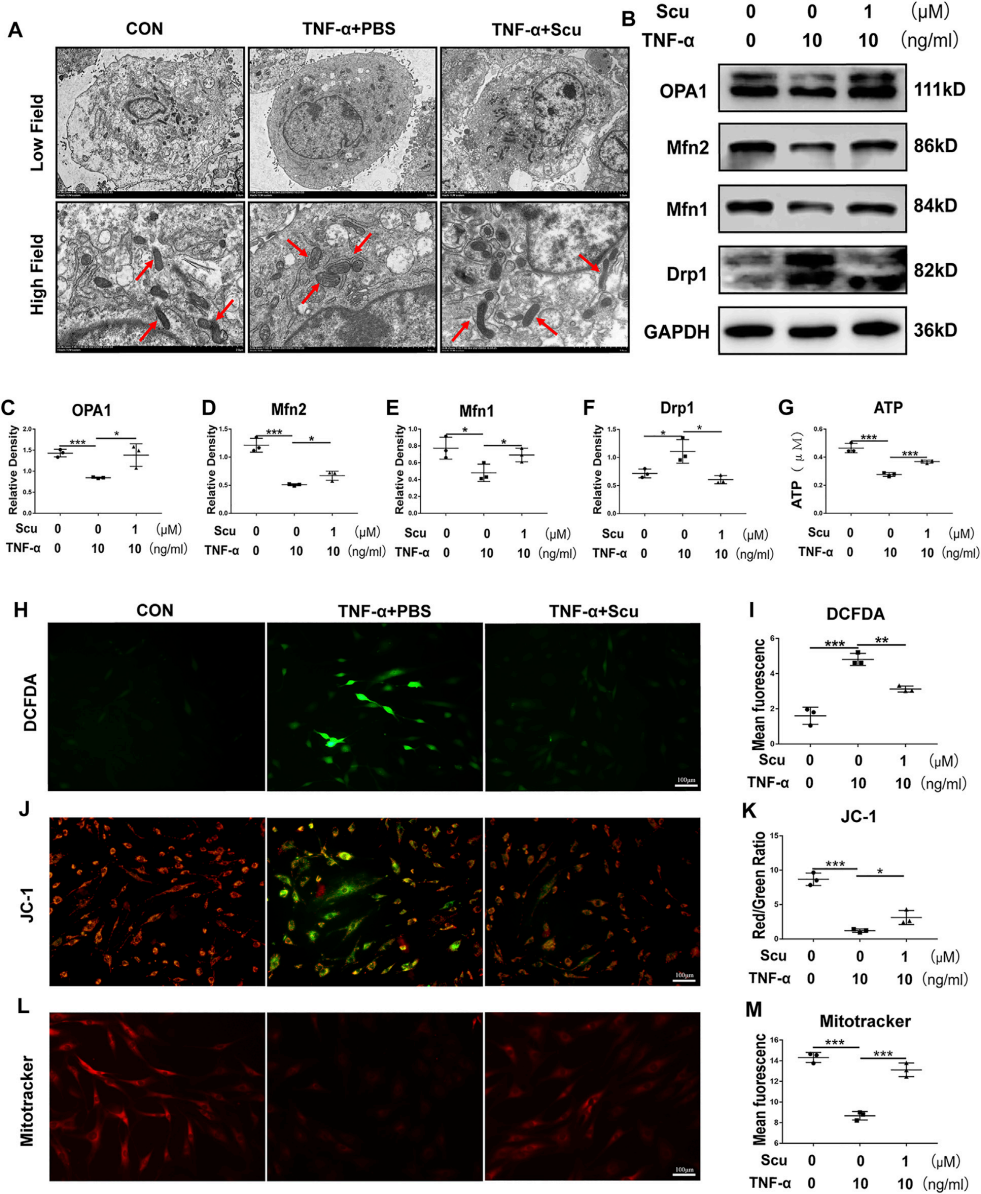
Scutellarin reversed oxidative stress and mitochondrial dysfunction and reduced ATP production induced by TNF-α in HNPCs. **(A)** HNPCs were cultured with PBS, TNF-α (10 ng/ml), TNF-α (10 ng/ml) +scutellarin (1 μM) for 24 h. The mitochondrial morphology of HNPCs was observed using TEM (scale bar: 5 μm). **(B–F)** After treatment as indicated for 48 h, total protein was extracted from each group. The protein levels of OPA1, Mfn1, Mfn2, and Drp1 in each group were detected by Western blotting. **(G)** ATP levels of HNPCs in each indicated group were examined. **(H,I)** ROS levels in HNPCs in each indicated group were detected with DCFDA (scale bar: 100 μm). **(J,K)** JC-1 was used to detect the mitochondrial membrane potential of HNPCs in each indicated group (scale bar: 100 μm). **(L,M)** MitoTracker was used to detect the mitochondrial membrane potential of HNPCs treated as indicated (scale bar: 100 μm). Each experiment was performed three times independently. (*p < 0.05, **p < 0.01, ***p < 0.001).

Exacerbation of oxidative stress and dysfunction of mitochondria are known to be harmful to inflammation and metabolism (Zhao et al., 2020); together with the finding of this study that scutellarin protects against inflammation and metabolism in NP cells, we were prompted to study whether the role of scutellarin is related to these detrimental factors. Herein, HNPCs were cultured, and transmission electron microscopy (TEM) was performed to observe the morphology of mitochondria. As shown in Figure 4A, the mitochondria were swollen and exhibited disrupted mitochondrial cristae following stimulation with TNF-α. However, treatment with scutellarin reversed this disordered alteration in mitochondrial morphology, which proved that scutellarin might be protective against mitochondrial damage in HNPCs. OPA1, Drp1, Mfn1, and Mfn2 are biomarkers of mitochondrial function (Zhao et al., 2020), all of which displayed a disorganized expression pattern after TNF-α stimulation, as assayed by Western blotting. Intriguingly, scutellarin treatment remarkably reversed the production of these molecules, suggesting the beneficial effect of scutellarin on mitochondrial function (Figures 4B–F) and ATP production. (Figure 4G). To further investigate the potential connection between scutellarin and mitochondrial ROS in HNPCs during IVDD, DCFDA, JC-1, and MitoTracker staining assays were performed to detect the production of ROS, mitochondrial membrane potential, and distribution pattern of mitochondria. As revealed in Figures 4H–M, our results further proved that scutellarin reversed TNF-α-induced mitochondrial dysfunction and oxidative stress in HNPCs.

### Scutellarin Improved the Degeneration of Intervertebral Disc in a Rat Intervertebral Disc Degeneration Model

**FIGURE 5:**
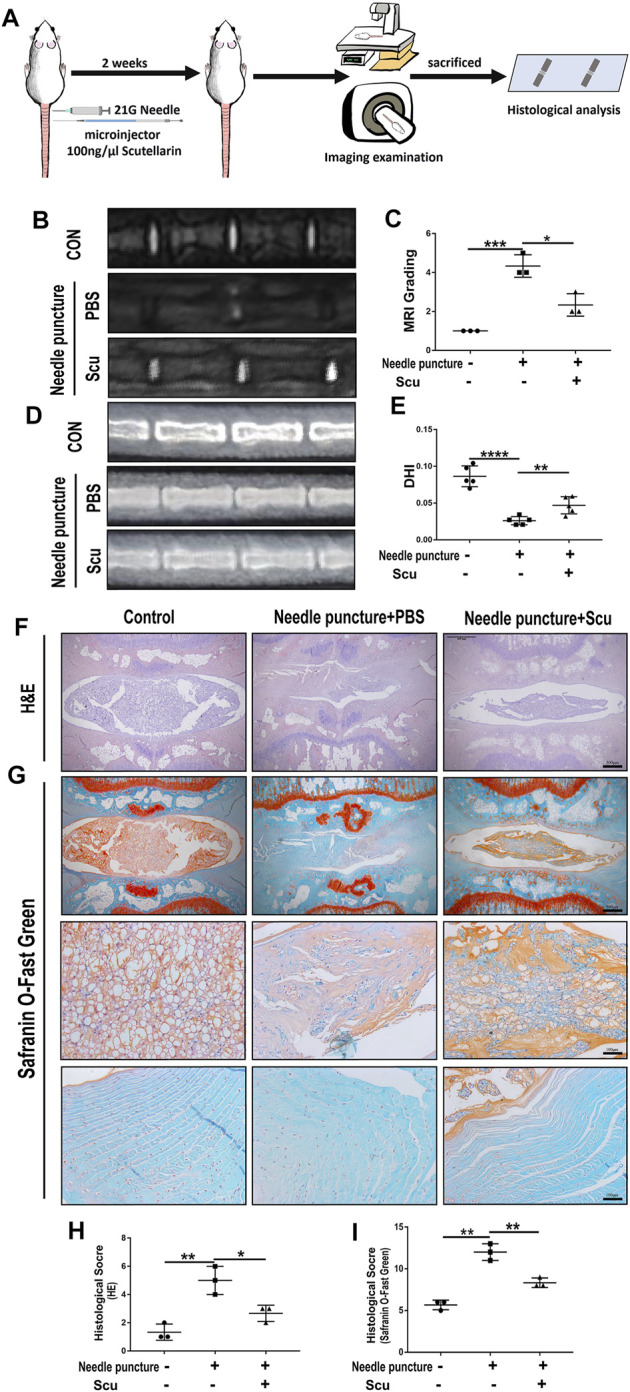
Scutellarin improved the degeneration of IVD in a rat IVDD model. **(A)** Schematic of the *in vivo* experiment and coccygeal disc needle puncture injections. **(B,C)** MRI to assess the degree of IVDD of Co6/7, 7/8, 8/9 in rats from the con group (*n* = 5), needle puncture + PBS injection group (*n* = 5) and needle puncture + scutellarin injection group (*n* = 5). **(D,E)** X-ray was obtained to measure the height of the intervertebral space of Co6/7, 7/8, and 8/9 in rats from each indicated group. **(F–I)** HE and Safranin O staining were performed to evaluate the cellularity and morphological changes of Co6/7, 7/8, 8/9 NPs in rats from each group. Then, the histological scores of each indicated group were calculated according to the grading scale previously published (scale bar: 500 μm or 100 μm). Each experiment was performed three times independently. (*p < 0.05, **p < 0.01, ***p < 0.001, ****p < 0.0001).

**FIGURE 6:**
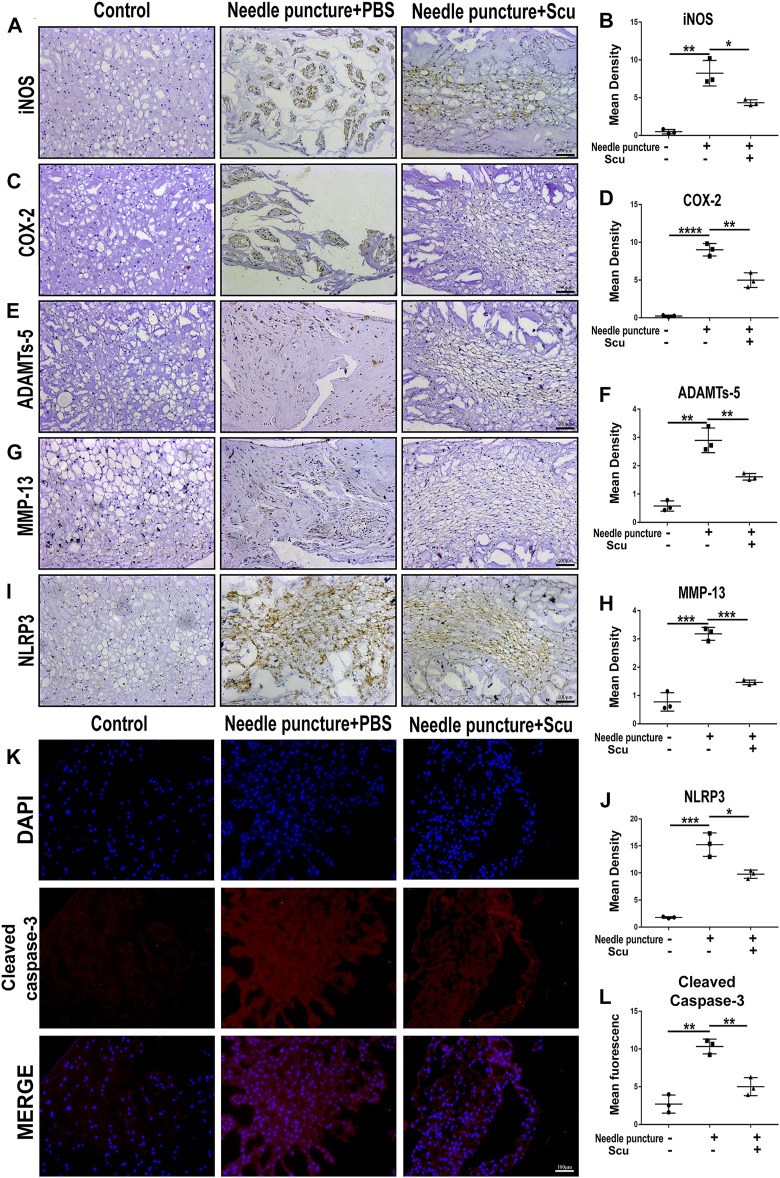
Scutellarin alleviates the degeneration of IVD in a rat IVDD model. **(A–J)** The expression levels of iNOS, COX-2, ADAMTS-5, MMP-13, and NLRP3 in IVD tissues from the con group (*n* = 5), needle puncture + PBS injection group (*n* = 5) and needle puncture + scutellarin injection group (*n* = 5) were detected by immunohistochemistry (scale bar: 100 μm). **(K,L)** The expression levels of cleaved caspase-3 in IVD tissues from each indicated group were assayed by immunofluorescence staining (scale bar: 100 μm). Each experiment was performed three times independently. (*p < 0.05, **p < 0.01, ***p < 0.001).

To investigate the role of scutellarin in IVDD *in vivo*, a rat IVD needle puncture model was established, and local delivery of scutellarin was performed through intradiscal injection (Figure 5A) as we have previously reported (Zhao et al., 2020; Wu et al., 2021; Yu et al., 2021). To detect the IVD signal, MRI-T2WI analysis was performed, which revealed higher signal intensity in the rat IVD following scutellarin treatment than in the PBS group following needle puncture, suggesting the improvement of IVDD (Figures 5B,C). In addition, an X-ray was performed for each group of IVD samples, the IVD height was diminished after needle puncture, and the IVD height was largely retained following scutellarin treatment (Figures 5D,E). Thereafter, the IVD samples were collected, followed by histological analysis. Figures 5F–I indicate that the morphological degeneration score of the IVD based on HE and Safranin O staining in this IVDD model was alleviated by treatment with scutellarin. Moreover, Safranin O staining showed that scutellarin reduced proteoglycan loss during the process of IVDD. To further study the degeneration-associated biomarkers in each group, IHC for the inflammatory factors iNOS and COX-2 (Figures 6A–D) and the metabolism molecules ADAMTS-5 and MMP-13 (Figures 6E–H) was performed, which showed that scutellarin greatly reduced the expression of these biomarkers in the rat IVDD model. Moreover, an IHC analysis of NLRP3 expression levels was performed, and Figures 6I,J indicate that scutellarin alleviated the production of NLRP3 in the IVDD rat model. Furthermore, immunofluorescence was performed, and as shown in Figures 6K,L, scutellarin treatment reduced the expression of cleaved caspase-3 in IVDD.

### Scutellarin Inhibited the NF-κB/Mapk Signaling Pathways and Suppressed Activation of the NLRP3 Inflammasome in Human Primary Nucleus Pulposus Cells

**FIGURE 7:**
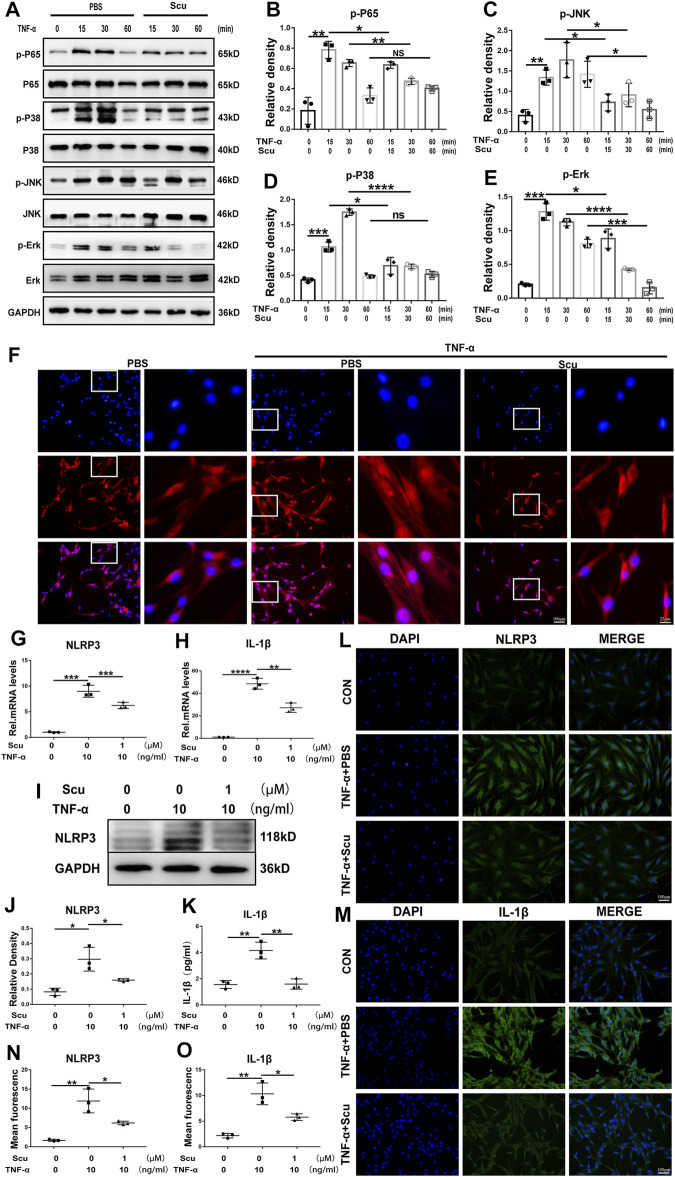
Scutellarin inhibited the NF-κB/MAPK signaling pathways and suppressed activation of the NLRP3 inflammasome in HNPCs. **(A–E)** HNPCs were treated with PBS, TNF-α (10 ng/ml), TNF-α (10 ng/ml) +scutellarin (1 μM) for 15 min, 30 min, and 1 h. Total protein was extracted, and the levels of p-p65, p65, p-p38, p38, p-JNK, JNK, p-ERK, and ERK were detected by Western blotting. **(F)** Nuclear translocation of p65 in HNPCs, which were treated as indicated for 6 h, was assayed through immunofluorescence staining (scale bar: 100 μm or 25 μm). **(G,H)** After culturing as indicated for 24 h, the mRNA expression of NLRP3 and IL-1β in each group was detected by real-time PCR. **(I,J)** Western blotting was performed to assay the protein levels of NLRP3 in each group treated for 48 h as indicated. **(K)** IL-1β secretion levels of each indicated group were detected by ELISA. **(L–O)** The expression levels of NLRP3 and IL-1β in HNPCs in the indicated groups were examined by immunofluorescence staining (scale bar: 100 μm). Each experiment was performed three times independently. (*p < 0.05, **p < 0.01, ***p < 0.001, ****p < 0.0001).

To date, the NF-κB and MAPK signaling pathways are known to facilitate the ageing process under several conditions and have been extensively studied in the development of IVDD. In this study, HNPCs were cultured and stimulated with TNF-α with or without scutellarin treatment. Thereafter, phosphorylation of critical parameters in the NF-κB signaling pathway p65 (Chen et al., 2019) and components of the MAPK signaling pathway (Li L. et al., 2021), including JNK, p38, and ERK1/2, were assayed through Western blotting. As shown in Figures 7A–E, phosphorylated p65 was enhanced by TNF-α, which was largely abolished by additional treatment with scutellarin. Moreover, Western blotting for p-JNK, p-p38, and p-ERK1/2 indicated that TNF-α activated all the mentioned MAPK signaling pathways, while scutellarin retained the activity of the p38, JNK, and ERK1/2 signaling pathways in cultured HNPCs. To further test the involvement of the NF-κB signaling pathway in the effect of scutellarin, IF staining was performed, and nuclear translocation p65 was assayed. Figure 7F indicates that TNF-α induced the nuclear translocation of p65, while scutellarin suppressed this change in HNPCs mediated by TNF-α.

The NLRP3 inflammasome is deeply associated with IVDD through the production of IL-1β as its downstream molecule and the induction of exaggerated cell death, including apoptosis (Chao-Yang et al., 2021). To determine whether NLRP3 is related to the function of scutellarin in HNPCs, mRNA and protein were isolated from HNPCs. Real-time PCR was performed for NLRP3 and IL-1β (Figures 7G,H), and Western blotting was performed for NLRP3 (Figures 7I,J), indicating that scutellarin administration attenuated the activation of NLRP3 inflammasome induced by TNF-α. Furthermore, the supernatant was collected from each group, and an enzyme-limled immunoassay (ELISA) was performed for IL-1β, which showed that TNF-α-mediated secretion of IL-1β in HNPCs was inhibited by scutellarin (Figure 7K). In addition, the cells of each group were collected, and IF staining for NLRP3 and its downstream molecule IL-1β was performed. As revealed in Figures 7L–O TNF-α enhanced the expression of NLRP3 and IL-1β in HNPCs, and scutellarin treatment significantly suppressed this trend.

## Discussion

Enhanced production of proinflammatory molecules is observed during the pathogenesis of IVDD (Lee et al., 2021). Various proinflammatory cytokines and chemokines have been reported to be associated with IVDD (De Luca et al., 2020; Jacobsen et al., 2020), among which TNF-α has been considered a predominant factor and shown to play a detrimental role in IVDD development (Wang et al., 2018). Previous studies have shown that TNF-α promotes the production of downstream inflammatory cytokines, leads to increased catabolism of the IVD, and exacerbates ECM degradation (Ashraf et al., 2021), which has become a promising target for IVDD therapy in the clinic.

As a critical proinflammatory molecule, TNF-α is located at the top of the inflammation cascade, which can facilitate a chain of inflammatory reactions by binding to its specific receptors (Tang et al., 2011). In our study, HNPCs were stimulated with TNF-α to investigate the role of scutellarin in inhibiting TNF-α-mediated inflammation during IVDD. COX-2 and iNOS are inflammation-associated biomarkers that are involved in IVDD and can be induced by TNF-α (Zhao et al., 2020). The expression levels of COX-2 and iNOS secreted by HNPCs were enhanced following stimulation with TNF-α, which was in line with previous findings. Intriguingly, after treatment with scutellarin, the expression pattern of these inflammatory cytokines was reversed compared with the expression pattern of these inflammatory cytokines in the TNF-α control group, suggesting that scutellarin might attenuate the inflammatory reaction of NP cells during IVDD.

Previous studies have shown that TNF-α leads to disorganization of cell metabolism in IVD, including reduction of Col-2 as well as aggrecan, and elevation of MMPs and ADAMTSs, which contribute to the destruction of the ECM and pathogenesis of IVDD (Liu Y. et al., 2021; Zhao and Li, 2021). TNF-α promotes the expression levels of catabolic markers, including MMP-13 and ADAMTS-5, which are harmful to ECM components during IVDD. Scutellarin alleviates Col-2 and aggrecan loss in chondrocytes during the development of osteoarthritis (Luo et al., 2020). In the present study, treatment with scutellarin alleviated the loss of Col-2 and aggrecan mediated by TNF-α and diminished the expression of MMP-13 and ADAMTS-5, implying that scutellarin might play a protective role in the disorganization of metabolism in IVDD.

Apoptosis causes a remarkable decrease in living and functional IVD cells, which is usually detected and extensively studied during the pathogenesis of IVDD (Xie et al., 2021). Studies have shown that TNF-α increases the apoptotic rate and senescence in NP cells, which has been considered a potential target for the investigation of IVD degeneration (Zhang et al., 2020). The disordered expression pattern of Bax and Bcl-2 is involved in the mitochondria-associated apoptosis pathway during the development of IVD degeneration, which enhances the expression of cleaved caspase-3 and promotes the apoptosis of NP cells (Lin et al., 2021). In the current study, TNF-α stimulation of HNPCs resulted in increased expression of Bax and cleaved caspase-3 and diminished levels of Bcl-2, which suggests that TNF-α causes disordered apoptosis in HNPCs by increasing the expression of proapoptotic parameters and reducing the expression of antiapoptotic parameters and may be involved in the mitochondrial apoptosis pathway. Intriguingly, treatment with scutellarin retained the expression levels of Bax, cleaved caspase-3, and Bcl-2. Moreover, TUNEL and live/dead cell assays indicated that scutellarin greatly repressed the occurrence of cell death upon stimulation with TNF-α, in line with our flow cytometry finding that a lower ratio of TNF-α-induced HNPC apoptosis was detected in the presence of scutellarin. This set of experiments suggested that scutellarin can alleviate apoptosis during the pathogenesis of IVDD.

Previous studies have reported the impaired morphology and dysfunction of mitochondria in nucleus pulposus cells during ageing (Ding et al., 2012), and mitochondrial dysfunction plays a detrimental role in the development of IVDD. In this study, TNF-α stimulation of NP cells led to damage to the mitochondrial structure, which was swollen and deformed, together with the abnormal alteration of mitochondrial functional biomarkers, including HNPCs OPA1, Drp1, Mfn1, and Mfn2, as previously reported (Xu et al., 2019). Furthermore, the production of ROS was enhanced, the mitochondrial membrane potential was impaired, and the distribution of mitochondria was disorganized by TNF-α, which was in line with previous findings. Interestingly, scutellarin reversed these changes in mitochondria and ROS production, and scutellarin antagonized disordered inflammatory reactions, metabolism, and apoptosis in HNPCs, suggesting that mitochondrial ROS might contribute to the mechanisms of scutellarin in IVDD.

The needle puncture rat IVDD model is widely used for investigating IVD degeneration *in vivo* (Cheng et al., 2021; Wu et al., 2021). The degenerative phenotype of IVD is displayed in this model, including alteration of disc signal in MRI, a decrease of disc height through X-ray, and loss of ECM component as well as disorganized expression of metabolic markers through histological analysis (Zhao et al., 2020). In the current study, intradiscal injection of scutellarin retained the signal and height of the IVD, attenuated the loss of major ECM content, and improved metabolism in IVDD, implying the beneficial effect of scutellarin to establish an antiageing microenvironment in IVD tissue.

Previous findings indicated that the role of TNF-α in IVDD is facilitated mainly by the activation of NF-κB as well as the MAPK signaling pathway (Lv et al., 2021). Activation of the NF-κB signaling pathway can accelerate IVDD, while antagonization of the NF-κB signaling pathway can alleviate the ageing process (Zhao et al., 2015). In this study, HNPCs experiments showed that scutellarin inhibited the activation of the NF-κB signaling pathway caused by TNF-α. Levels of the critical parameter for the NF-κB signaling pathway, phosphorylated p65 (p-p65) proteins were increased after treatment with TNF-α. Intriguingly, the levels of p-p65was significantly reduced by additional scutellarin treatment. Moreover, nuclear translocation of p65 was promoted by TNF-α, indicating the activation of the NF-κB signaling pathway (Liu Z. et al., 2021). However, this change was suppressed by scutellarin treatment. This set of experiments suggests that scutellarin is effective in blocking the activation of the NF-κB signaling pathway during IVDD.

Another critical signaling pathway associated with degeneration is the mitogen-activated protein kinase (MAPK) signaling pathway (Lv et al., 2021). The MAPK signaling pathway has three types of signaling pathways, including JNK, p38, and ERK1/2, all of which are reported to be involved in IVDD (Huang et al., 2020). Herein, the activation of the three signaling pathways was assayed, indicating that TNF-α stimulation was connected with all of them. In this study, scutellarin reversed the p38, JNK, and ERK1/2 signaling pathways, suggesting that the role of scutellarin in IVDD might also rely on its interference with the MAPK signaling pathway.

**FIGURE 8:**
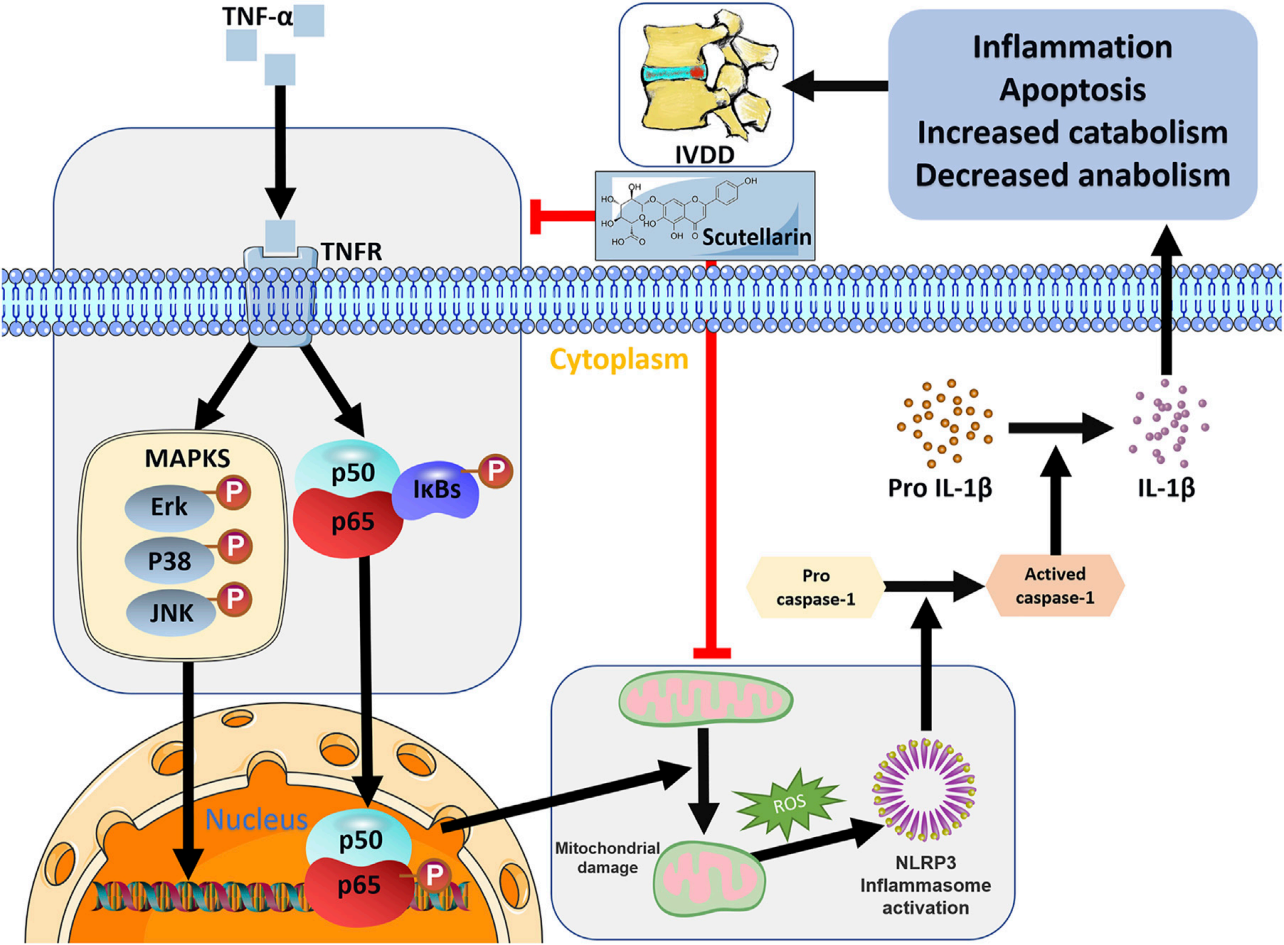
Schematic depicting a proposed model for the function of scutellarin in intervertebral disc degeneration.

The NLRP3 inflammasome is harmful to the metabolism and apoptosis of NP cells and plays a detrimental role in IVD degeneration (Sun et al., 2021). Mitochondria of cells contribute to the activation of the NLRP3 inflammasome through several mechanisms, among which mitochondrial dysfunction and mitochondrial ROS generation are predominant factors (Zhao et al., 2020; Sun et al., 2021). In this study, we found exaggerated mitochondrial dysfunction and enhanced levels of NLRP3 and IL-1β induced by TNF-α, suggesting enhanced mitochondrial ROS-dependent NLRP3 activation in HNPCs, which was markedly repressed by scutellarin treatment. The NLRP3 inflammasome triggers the secretion of active IL-1β, further facilitating the progression of IVD degeneration (Zhou et al., 2021). In our study, NLRP3 expression levels and IL-1β secretion were elevated in HNPCs following TNF-α stimulation, while scutellarin treatment reversed this trend, implying the effect of scutellarin on the maintenance of normal activation of the NLRP3 inflammasome during IVDD. In conclusion, scutellarin exerts a protective effect on alleviating IVDD by repressing the inflammatory response of HNPCs, reducing the loss of major ECM components, and preserving mitochondrial function by reducing ROS production, which reduces the activation of the NLRP3 inflammasome and the expression of apoptosis-associated markers (Figure 8). In our future studies, we expect to develop scutellarin as a potential therapeutic tool for IVDD.

## Data Availability

The original contributions presented in the study are included in the article/Supplementary Materials, further inquiries can be directed to the corresponding author
