# Supplementary material for: Scutellarin Protects Against Mitochondrial Reactive Oxygen Species-Dependent NLRP3 Inflammasome Activation to Attenuate Intervertebral Disc Degeneration
Source: Front Bioeng Biotechnol. 2022 Aug 11;10:883118. doi: 10.3389/fbioe.2022.883118 (PMC9403485; doi:10.3389/fbioe.2022.883118)
Supplement: Supplementary file 1 [file DataSheet1.docx]

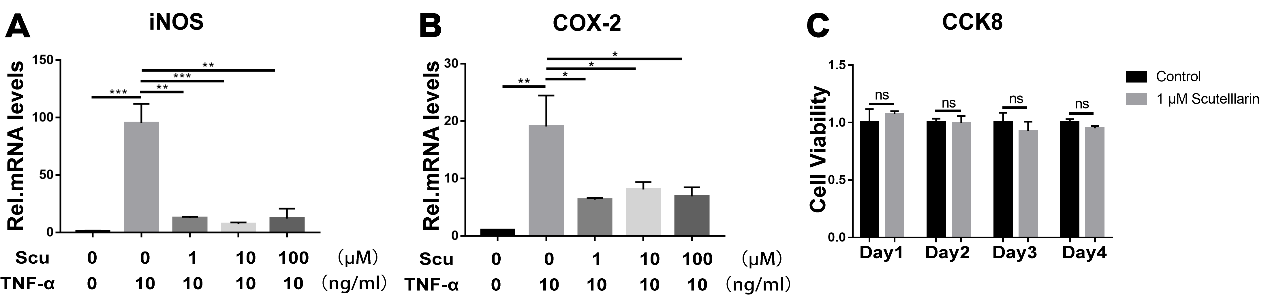


Fig. 1: **(A-B)** HNPCs were treated with or without TNF-α (10 ng/ml) in the absence or presence of scutellarin (1 μM, 10 μM, 100 μM) for 24 hours. Total RNA from each group was extracted, and real-time PCR was performed to examine the mRNA expression of iNOS and COX-2. **(C)** HNPCs were treated with or without scutellarin ((1 μM, 10 μM, 100 μM) for 1, 2, 3 and 4 days. Cell viability assay performed by CCK8 test.


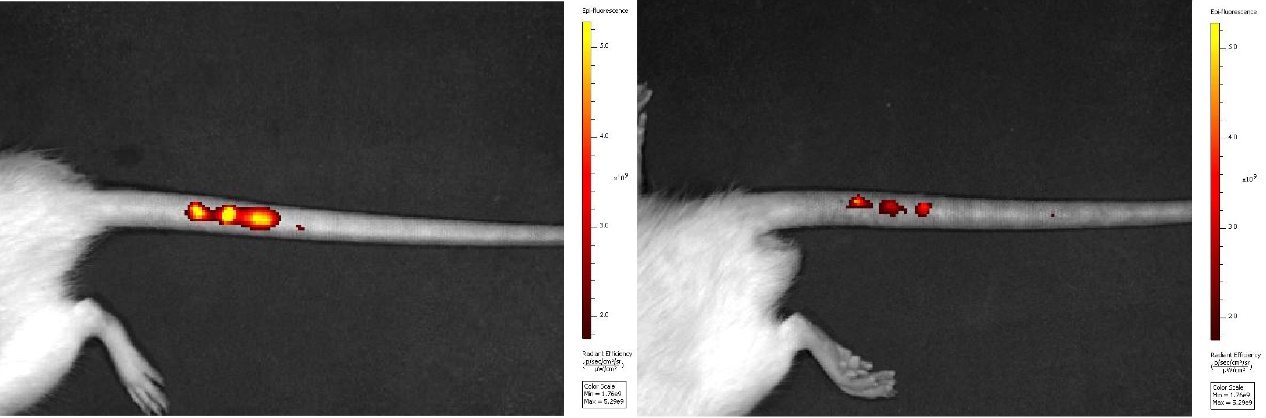


Fig. 2: 2.5 μl fluorescent small molecule solution was injected into the 7/8, 8/9, 9/10 coccygeal disc. The fluorescence signal intensity of the discs was detected by the intravital fluorescence imaging system right now and 1day after injection.


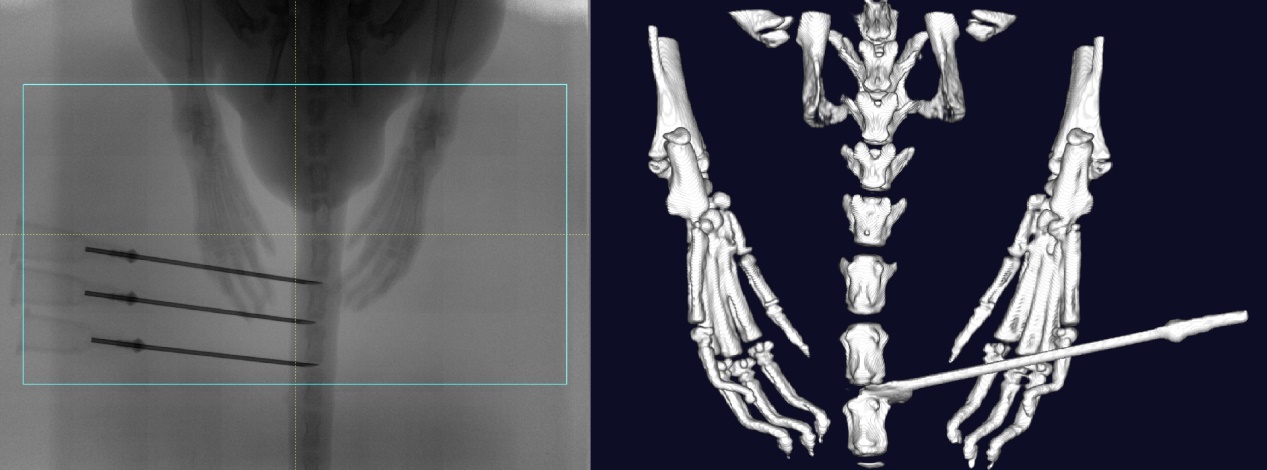


Fig. 3: The 6/7, 7/8, 8/9 coccygeal discs were located by X-ray and CT before the establishment of rat needle puncture model.


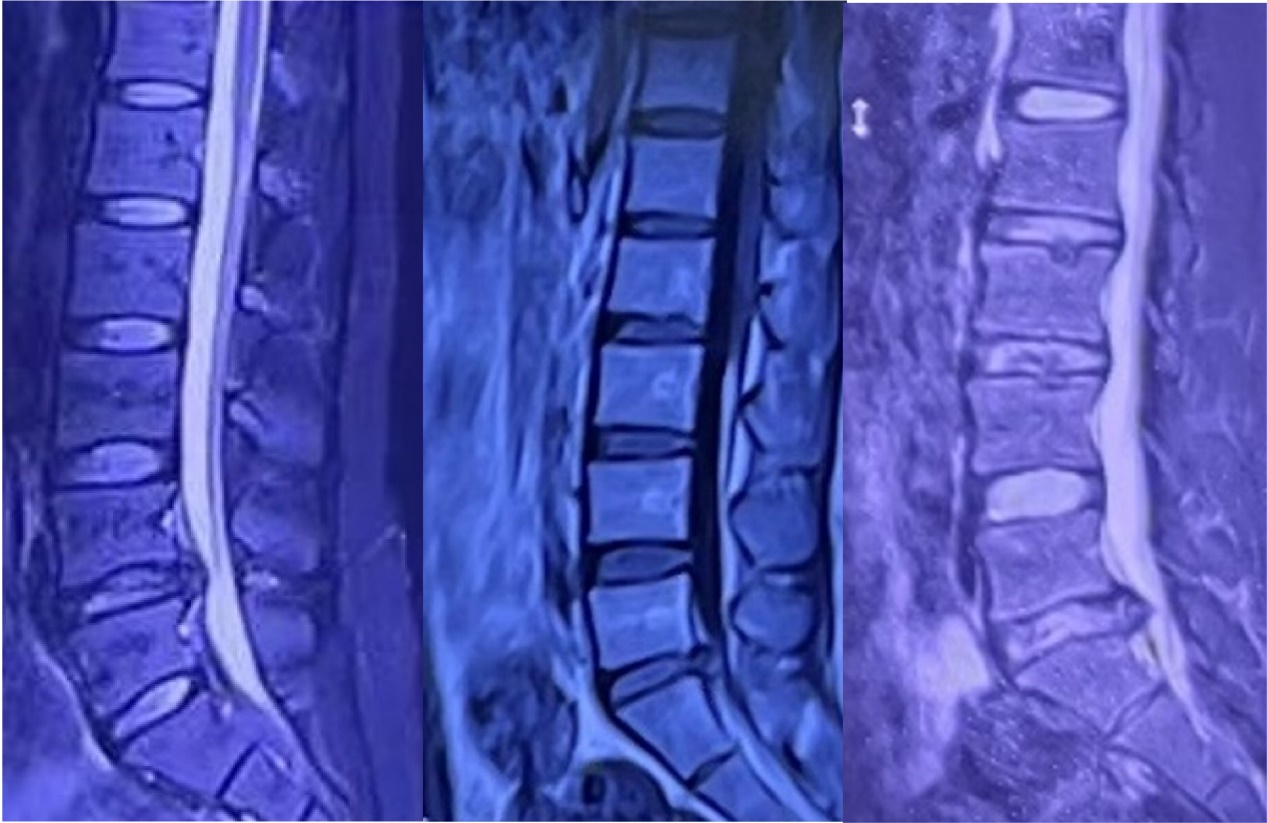

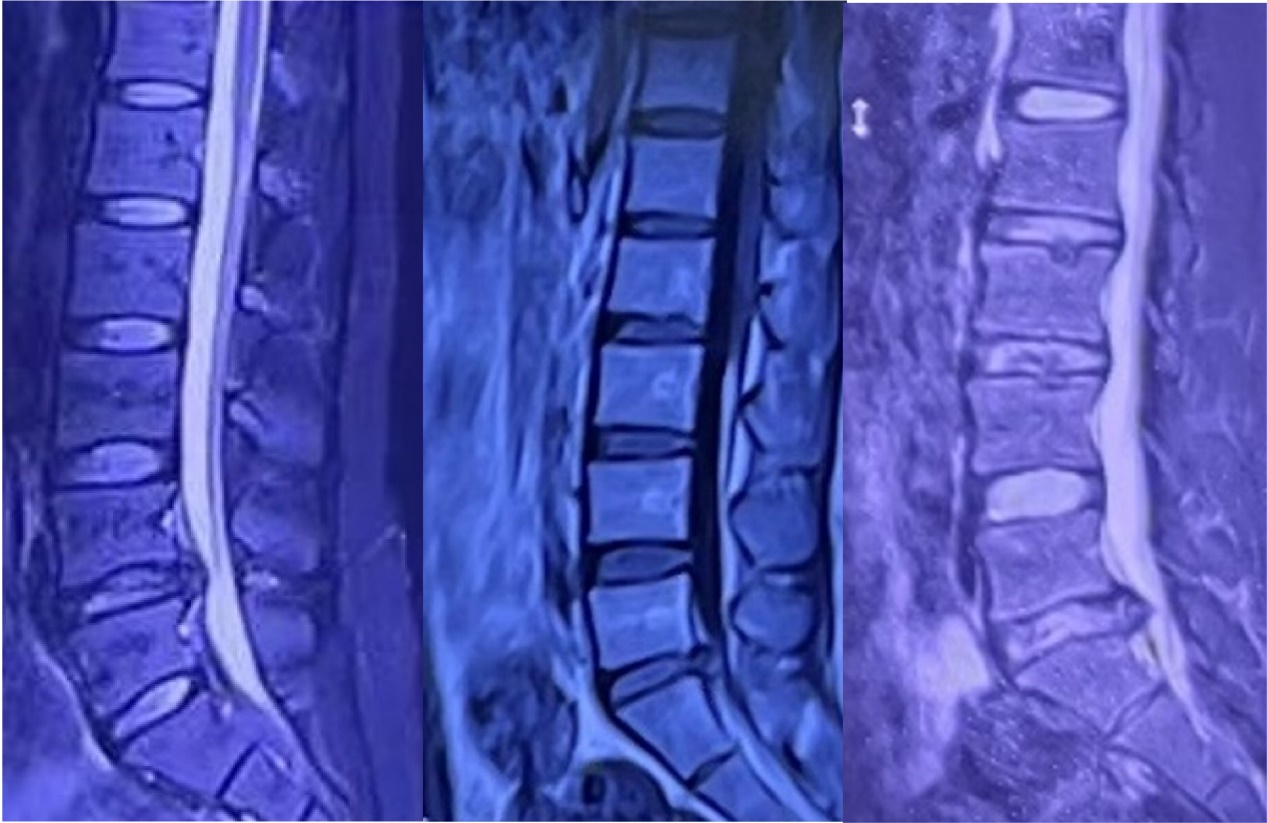


Fig. 4: MRI images of three patients undergone surgical treatment at Qilu Hospital with secondary disc degeneration.
